# Pharmacological effects of *Salvia miltiorrhiza *(*Danshen*) on cerebral infarction

**DOI:** 10.1186/1749-8546-5-22

**Published:** 2010-06-21

**Authors:** Tsai-Hui Lin, Ching-Liang Hsieh

**Affiliations:** 1 Department of Chinese Medicine, China Medical University Hospital, Taichung, 40402, Taiwan; 2 Graduate Institute of Acupuncture Science, China Medical University, Taichung, 40402, Taiwan; 3 Acupuncture Research Center, China Medical University, Taichung, 40402, Taiwan

## Abstract

*Danshen*, the dried root of *Salvia miltiorrhiza*, is a Chinese medicine used to promote blood flow and treat vascular disease. The present article reviews the pharmacological effects of *Danshen *on cerebral infarction and possible interactions between *Danshen *and Western drugs. *Danshen *may reduce or prolong the development of atherosclerosis and may have anti-hypertensive and anti-platelet aggregation effects, which prevent cerebral infarction. *Danshen *may enhance endogenous anti-oxidative enzyme activities such as the expression of endothelial nitric oxide synthase and may scavenge oxygen free radicals. Prevention and treatment of cerebral infarction by *Danshen *involves multiple pathways, including anti-atherosclerosis, anti-hypertension, anti-platelet aggregation, anti-inflammatory and anti-oxidative effects.

## Review

### Background

*Danshen*, the dried root of *Salvia miltiorrhiza*, is used in Chinese medicine to treat vascular disease. According to Chinese medicine theory, *Danshen *promotes blood flow and resolves blood stasis. Among stroke patients, 80% suffer from cerebral infarction and 20% cerebral hemorrhage [1]. Cerebral infarction is an ischemic condition of the brain including thrombosis, embolism or systemic hemodynamic hypotension. Cerebral infarction is often caused by atherosclerosis of large and small arteries. The etiology of atherosclerosis and stroke is related to inflammation and genetic factors. Ischemic cerebral infarction may be prevented through anti-inflammation and treatment for vascular diseases, heart diseases and hypertension [2-4]. Anti-thrombosis and thrombolysis are used to treat ischemic cerebral infarction [2]. The most effective method to re-establish cerebral blood flow is thrombolytic therapy; however, this therapy is often at the risk of bleeding [2]. Neuroprotection is also a potential treatment [5].

While *Danshen *or its ingredients reduce the infarction volume, the action mechanism of *Danshen *remains obscure [6-8]. *Danshen *contains the lipid-soluble tanshinone I (Tan I), tanshinone IIA (Tan IIA), cryptotanshinone and dihydrotan-shinone as well as the water-soluble danshensu and salvianolic acid B (Sal B) [9]. *Danshen *inhibits platelet aggregation and promotes fibrinolysis [10,11].

Covering literature between 1990 and 2009, this article reviews basic and clinical studies on *Danshen *in the prevention and treatment for cerebral infarction. The key words used for search in the PubMed library were '*Danshen*' or '*Salvia miltiorrhiza*' combined with 'cerebral infarction', 'ischemic' or 'cerebral ischemia'.

### Prevention of cerebral infarction

#### Anti-atherosclerosis and anti-inflammatory effects

Atherosclerosis is caused by endothelial damage, which may lead to platelet aggregation and the release of platelet factor, resulting in the proliferation of smooth muscle in the arterial intima. The process of atherosclerosis comprises inflammation and involves both innate and adaptive immunity [12]. The tanshinones components of *Danshen *(tanshinone I, dihydrotanshinone and cryptotanshinone) inhibit the production of interleukin-12 (IL-12) induced by lipopolysaccharide (LPS)-activated macrophages and the production of interferon-γ induced by keyhole limpet hemocyanin-primed lymph node cells [13]. Tanshinones also inhibit the expression of the IL-12 p40 gene and prevent nuclear factor-κB (NF-κB) from binding to κB site [13]. As IL-12 and NF-κB are closely associated with inflammatory responses, tanshinones may have anti-inflammatory effects [13]. Tumor necrosis factor-α (TNF-α), a pro-inflammatory cytokine, is regulated by NF-κB while vascular adhesion molecule-1 (VCAM-1) regulates the migration of leukocytes into the vessel wall. Pre-treatment with either aqueous ethanolic extract of *Danshen *or Sal B component of *Danshen *inhibits TNF-α-induced expression of VCAM-1 and TNF-α-induced activation of NF-κB in human aortic endothelial cells, suggesting that both SME and Sal B posses anti-inflammatory properties and are closely associated with atherogenesis because leukocytes migrate into the vessel wall in the early stage of atherogenesis [14]. Aqueous extract of *Danshen *suppresses the adhesion rate of neutrophils stimulated by TNF-α, and also inhibits adhesion of neutrophils stimulated by *N*-formyl-methionyl-leucyl-phenylalanine (fMLP) [15]. Aqueous extract of *Danshen *inhibits TNF-α-induced up-regulation of E-selectin, intracellular molecule-1 (ICAM-1) and VCAM-1 expression [15]. *Danshen *treatment lowers plasma viscosity, erythrocyte aggregation and fibrinogen levels in rats with traumatic brain injury [16]. Tan IIA increases estrogen receptor activity in HeLa cells. Tan IIA inhibits iNOS (inducible nitric oxide synthase) protein production and nitric oxide (NO) production and inhibits pro-inflammatory cytokine IL-1β, IL-6 and TNF-α via estrogen receptors in lipopolysaccharide (LPS) activated RAW 264.7 macrophages, suggesting that Tan IIA may serve as an estrogen-receptor-like modulator to produce immune responses [17]. C-reactive protein (CRP), an inflammation marker, induces pro-inflammatory cytokines through NF-κB and contributes to atherosclerosis in endothelial cells [18,19]. Tan IIA inhibits NF-κB-DNA complex, NF-κB binding activity and the phosphorylation of IκBα and inhibit the translocation of NF-κB from cytosol to nuclei, demonstrating that tanshinone possesses anti-inflammatory properties [20]. Tan IIA down-regulates protein expression and activities of matrix metalloproteinase-2 and -9 (MMP-2, MMP-9) and reduces the VCAM-1 and IL-1β levels to suppress the increase in the aorta intimal area in rabbits treated with a high-fat-diet [21]. Triterpenoids-enriched extract of *Danshen *reduces the aortic atherosclerotic lesion area and inhibits inflammatory serum marker of CRP and monocyte chemotactic protein (MCP-1) [22]. Moreover, triterpenoids-enriched extract of *Danshen *reduces the serum levels of total cholesterol and triglyceride in low density lipoprotein receptor (LDLR) ^+^mice via the inhibition of the inflammatory markers CRP and MCP-1 [22]. Triterpenoids are anti-atherogenic which may be associated with its anti-inflammatory properties [22]. In summary, the anti-atherosclerosis and anti-inflammatory actions of *Danshen *play roles in the prevention of cerebral infarction.

#### Anti-hypertensive effects

Hypertension is a risk factor for cerebral infarction. Maintaining blood pressure within the ideal range has recently been recognized as an important principle in the prevention and treatment of ischemic cerebral infarction. Aqueous extract of *Danshen *alleviates hypertension in the two-kidney/one-clip (2K1C) Goldblatt renovascular hypertensive rats model and reduced the activity of serum angiotensin converting enzyme and the levels of serum aldosterone [23,24]. MTB70 is composed of 70% (70 mg/ml) of Magnesium tanshinoate B (MTB) which is one of the aqueous active components of *Danshen*. MTB70 (0.7-175 mg/kg) reduces blood pressure more than the aqueous extract of *Danshen *with same volume in a saline- or phenylephrine (2.5 μg/kg bolus injection and followed by 100 μg/kg at 0.13 ml/hr injection continuously) -induced hypertension model in rats; However this rapid and potent anti-hypertensive effect is short-lived [25]. Tanshinone IIA reduces blood pressure in the 2K1C renovascular hypertension model of hamsters [26]. Overall, the anti-hypertensive effects of *Danshen *include inhibition of angiotensin converting enzyme and generation of eNOS and/or vasodilatation.

### Treatment of cerebral infarction

#### Anti-platelet aggregation effects

Anti-platelet aggregation, which involves platelet adhesion, is widely used to treat acute ischemic cerebral infarction [27]. Platelet adhesion increases in mice with middle cerebral artery occlusion (MCAo) [28]. Eight derivatives of *Danshen *inhibit platelet aggregation induced by adenosine diphosphate (ADP) *in vitro *in rabbit plasma [29]. Salvianolic acid B (SAB) inhibits platelet deposition to collagen at venous and arterial shear rates, suggesting that SAB inhibits platelet adhesion to collagen via interfering collagen receptor α2β1 [30]. Salvianolic acid inhibits ADP-induced platelet aggregation in platelet-rich plasma and in washed platelets both *in vivo *and *in vitro*, probably through changing protein expression [31]. *Danshen *decreases the malondialdehyde (MDA) levels of platelets and increases the superoxide dismutase (SOD) activity of platelets to inhibit platelet aggregation in pulmonary thromboembolism induced by collagen and adrenaline in mice [32]. The 764-3 (100 μg/ml), purified compound of *Danshen *extract, inhibits platelet aggregation induced by arachidonic acid or ADP in humans and rabbits [33]. *Danshen *decreases the concentration of intra-platelet free calcium, which is closely associated with platelet aggregation and release [34]. Overall, *Danshen *exhibits anti-platelet aggregation effects via multiple pathways, e.g. the inhibition of intra-platelet calcium and anti-oxidant activities.

#### Neuroprotection through anti-inflammatory effects

Inflammatory responses are critically important to a patient after brain ischemia/reperfusion injury. The pro-inflammatory cytokines IL-1β, TNF-α and IL-6 increase after an ischemia attack, which enhances the expression of adhesion molecules including ICAM-1 and P-selectin, leading to brain edema and neuronal death [35,36]. In addition, leukocytes, neurons and activated microglial cells in ischemic damaged region release cytokines, chemokines and oxygen free radicals which lead to secondary brain tissue damage. Matrix metalloproteinases, which cleave protein components of the extracellular matrix, play a role in neurovascular remodeling and neuronal death [35,36]. The microglia may be activated during brain damage including ischemia, inflammation and infection [37]. The increases of activated microglia may represent the severity of neuronal damage in a MCAo rat model [38]. *Danshen *inhibits superoxide generation by microglia in primary microglia cell cultures from rat brains [39]. *Danshen *reduces CD18 and CD11b immunoreactive cells in the peri-vascular region and inhibits leukocyte infiltration and neuronal death in the cerebral infarction region in the ischemia-reperfusion MCAo rat model. As leukocytes are mediated via the combination of surface receptors of CD18 and CD11b and intracellular adhesion molecules of endothelium, the neuroprotection capability of *Danshen *may be associated with the inhibition of leukocyte adherence to the endothelium [40]. A study on the effects of *Danshen *on inflammatory response in cerebral ischemia and reperfusion injury in the MCAo rat model indicates that pre-treatment with Tanshinone IIA reduces the cerebral infarction area, neurological deficit score and cerebral edema. As pre-treatment with Tanshinone IIA reduces TNF-α, the myeloperoxidase (MPO) marker of leukocytes, E-selectin and ICAM-1 in ischemic brain tissue and serum IL-8, *Danshen *may inhibit inflammatory responses to reduce brain damage induced by ischemia-reperfusion injury in rats [41]. The *Danshen *dripping pill (DDP) is composed of *Danshen*, *Panax notoginseng *and *Dryobalanops camphor*. A controlled pilot study (106 patients) found that the recurrent rate in the DDP group was 9.6% compared with 24.1% in the non-DDP group. The serum CRP levels decreased from 2.33 to 1.50 mg/L in the DPP group were greater than those decreased from 2.21 to 1.77 mg/L in the non-DDP group [42]. Overall, *Danshen *has anti-inflammatory activity and may improve cerebral infarction and provide neuroprotection.

#### Neuroprotection through anti-oxidative effects

Nitrogen and oxygen free radicals involved in the production of reactive oxygen species (ROS) play an important role in brain damage during the reperfusion period following ischemia [43,44]. These ROS, which are involved in brain damage after cerebral ischemia, affect the signal transmission of mitochondria and DNA repair of enzyme and transcription factors, leading to apoptosis [45]. *Danshensu *and salvianolic acid B are phenolic acids of *Danshen*, both of which have scavenging activities towards free radicals of hydroxyl, 1,1-diphenyl-2-picryl-hydrazyl (DPPH), 2-azino-bis(3-ehtylbenzthiazoline-6-sulfonic acid) (ABTS) free radicals, hydrogen peroxidase and superoxide anion in human umbilical vein endothelial cells [46]. Our previous studies show that *Danshen *reduces the cerebral infarction volume and the neurological deficit score and reduces luminal-chemiluminescence counts [8]. As luminal-chemiluminescence counts may represent ROS, these effects of *Danshen *may be associated with its free radical scavenging activities in ischemia-reperfusion injured rats [8]. Intra-abdominal administration of Tan IIB, a major active component of *Danshen*, reduces the focal infarct volume, neurological deficit and apoptosis in the MCAo rat model. As apoptosis is a major pathway leading to neuronal death, Tan IIB is neuroprotective [47]. Damage from oxygen free radicals occurs in the ischemic/reperfusion injury under oxidation response to cell membrane lipids, proteins or DNA. Inhibiting oxidation and free radical scavenging are important in the treatment of ischemic cerebral infarction. A study using a 4-vessel occlusion rat model [48,49] found that (1) *Danshen *reduced an increase of cerebral NO and MDA 30 minutes prior to occlusion and that (2) pre-treatment with *Danshen *increased a significantly decreased SOD activity. NO mediates glutamate neurotoxicity and the inhibition of NO synthase may prevent the development of brain edema. SOD is a scavenger of superoxide anion and the levels of MDA may reflect the degree of lipid peroxidation. Thus *Danshen *is neuroprotective in this occlusion and reperfusion rat model [48,49]. Isopropyl-β,-(3,4-dihydroxyphenyl)-α-hydroxypropanoate (ND-309), a metabolite of *Danshen *in rat brain, reduces cerebral infarction volume, brain edema and neurological deficit in the MCAo rat model. Moreover, ND309 reduces brain tissue MDA and increases the reduction of ATP induced by ischemia-reperfusion injury [50] of mitochondrial ATP, mitochondrial SOD and glutathione peroxidase (GSH-Px) activities induced by ischemia-reperfusion injury. ND-309 is neuroprotective role to reduce brain damage induced by cerebral ischemia [50]. *Danshen *reduces the neurological deficit score and the levels of MDA and increases SOD activities in the MCAo rat model [51]. Pretreatment with *Danshen *may reduce brain edema and MDA concentration of cerebral cortex and hippocampus region in an ischemia-reperfusion injured rat model while *Danshen *may increase levels of catalase, SOD, GSH and ATP of the cerebral cortex and hippocampus region [52]. In summary, *Danshen *demonstrates neuroprotective effects that are closely associated with anti-oxidative action.

### Drug interactions with warfarin

Warfarin is an anticoagulant used to prevent atrial fibrillation, valvular heart disease, ischemic stroke and deep venous thrombosis [53]. *Danshen *inhibits platelet adhesion and aggregation and suppresses the formation of thromboxane A _2 _[53]. The interaction between *Danshen *and warfarin can cause bleeding and prolong prothrombin time or the International Normalized Ratio (INR) [53]. Co-administration of *Danshen *and warfarin should be avoided or closely monitored [54]. Tanshinones inhibits CYP1A1, CYP2C6 and CYP2C11-mediated warfarin metabolism to increase the concentration of warfarin [55]. Aspirin prevents and alleviates cerebral infarction. *Danshen *displaces the binding between aspirin and protein, thereby increasing free aspirin concentration in serum [56]. Atrial fibrillation, which may be prevented and treated by digoxin, is closely related to cerebral infarction induced by embolus. *Danshen *has digoxin-like immunoreactivity leading to a false interference of digoxin concentration [56].

### Compared free radical scavenging ability with other herbs

The extracts of Ginkgo biloba L. leaves (Egb 761), with free radical scavenging activity, reduce the size of cerebral infarction and improve neurological behavior in rats with permanent and transient MCAo [57,58]. Egb 761 is widely used to treat ischemic cerebral infarction. The concentration of salvianolic acid B is lower than Egb 761 in scavenging activity of superoxide anion and hydroxyl radicals in rat microsome, and in SH-SY5Y induced by H _2_O _2 _[59]. *Panax notoginseng *(*Sanqi*) is also used to treat vascular disorders. *Danshen *is stronger than *Panax notoginseng *in scavenging activity of superoxide anion, hydroxyl and DPPH radicals, yet it is weaker than *Panax notoginseng *in the scavenging activity of hydrogen peroxide and ferrous ion chelating activity [60]. While *Sanqi *demonstrates strong ferrous ion chelating activity and strong scavenging activities of hydrogen peroxide and hydroxyl radicals, it is weak in the scavenging activities of superoxide anion and DPPH radicals. Therefore, the scavenging capabilities of *Danshen *and *Sanqi *are quite different among the various free radicals [60].

## Conclusion

Prevention and treatment of cerebral infarction by *Danshen *involves multiple pathways, including anti-atherosclerosis, anti-hypertension, anti-platelet aggregation, anti-inflammatory and anti-oxidative effects (Additional file 1).

## Abbreviations

Tan I: tanshinone I; Tan II: tanshinone II; Sal B: salvianolic acid B; LPS: lipopolysaccharide; NF-κB: nuclear factor-κB; TNF-α: tumor necrosis factor-α; VCAM-1: vascular adhesion molecule-1; fMLP: N-formyl-methionyl-leucyl-phenylalanine; ICAM-1: intracellular molecule-1; iNOS: inducible nitric oxide synthase; NO: nitric oxide; MMP-2, MMP-9: matrix metalloproteinase-2 and -9; MCP-1: monocyte chemotactic protein; LDLR: low density lipoprotein receptor; 2K1C: two-kidney/one-clip; SME: Salvia miltiorrhiza extract; MTB: magnesium tanshinoate B; MCAo: middle cerebral artery occlusion; ADP: adenosine diphosphate; MDA: malondialdehyde; SOD: superoxide dismutase; MPO: myeloperoxidase; DDP: Danshen dripping pill; ROS: reactive oxygen species; DPPH: 1,1-diphenyl-2-picryl-hydrazyl; ABTS: 2-azino-bis(3-ehtylbenzthiazoline-6-sulfonic acid); ND-309: isopropyl-β,-(3,4-dihydroxyphenyl)-α-hydroxypropanoate; Egb 761: the extracts of Ginkgo biloba L. leaves

## Competing interests

The authors declare that they have no competing interests.

## Authors' contributions

THL searched the literature, organized the data and wrote the manuscript. CLH analyzed the data and revised the manuscript. Both authors read and approved the final version of the manuscript.

## Supplementary Material

Additional file 1 **Possible pharmacological actions of *Danshen *for prevention and treatment of cerebral infarction**. Supplemental table Click here for file
