# Supplementary material for: Pharmacological effects of Salvia miltiorrhiza (Danshen) on cerebral infarction
Source: Chin Med. 2010 Jun 21;5:22. doi: 10.1186/1749-8546-5-22 (PMC2910010; doi:10.1186/1749-8546-5-22)
Supplement: Additional file 1 — Possible pharmacological actions of Danshen for prevention and treatment of cerebral infarction. Supplemental table [file 1749-8546-5-22-S1.DOC]

**Additional file 1** Possible pharmacological actions of *Danshen* for prevention and treatment of cerebral infarction

| **Pharmacological actions** | | **References** |
| --- | --- | --- |
| **Anti-hypertension** |  |  |
|  | 1. *Danshen* can improve blood pressure and also can reduce the activity of serum angiotensin converting enzyme and the levels of serum aldosterone | [23,24] |
|  | 2. Tan IIA can reduce mean blood pressure from 161.2 to 130.0 mmHg, increase normalized arteriolar diameter from 1.0 to 1.25 and 1.57 | [26] |
| **Anti-platelet aggregation** |  |  |
|  | 1. Eight derivatives of *Danshen* may inhibit platelet aggregation *in vitro*. | [29] |
|  | 2. SAB can inhibit platelet deposition to collagen dose-dependently; directly inhibit the interaction between soluble α2β1 and collagen | [30] |
|  | 3. Salvianolic acid inhibit ADP-induced platelet aggregation in platelet-rich plasma and in washed platelets *in vivo* and *in vitro*. | [31]. |
|  | 4. *Danshen* can inhibit platelet aggregation in pulmonary thromboembolism induced by collagen and adrenaline in mice. | [32] |
|  | 5. The 764-3 component of *Danshen* extract can inhibit platelet aggregation induced by arachidonic acid or ADP in humans and in rabbits | [33] |
| **Anti-inflammation** |  |  |
|  | 1. *Danshen* can reduce CD18 and CD11b immunoreactive cells in the peri-vascular region, and also can inhibit leukocyte infiltration and neuronal death in the cerebral infarction region in an ischemia-reperfusion middle cerebral artery occlusion (MCAo) rat model | [40]. |
|  | 2. *Danshen* can reduce tumor necrosis factor-α (TNF-α), the myeloperoxidase (MPO) marker of leukocytes, E-selectin and intracellular adhesion molecule-1 (ICAM-1) in ischemic brain tissue, and serum interleukin-8 (IL-8) in ischemia-reperfusion injury rat model. | [41] |
|  | 3. *Danshen* dripping pill (DDP) containing *Panax notoginseng* and *Dryobalanops camphor* can decrease the recurrence rate of stroke/transient ischemic attack (TIA), and also can decrease serum C reactive protein (CRP) levels. | [42] |
| **Anti-oxidation** |  |  |
|  | 1. Danshensu and SAB have scavenging activities toward free radicals of hydroxyl, DPPH, ABTS, hydrogen peroxidase and superoxide anion in human umbilical vein endothelial cells. | [46] |
|  | 2. *Danshen* can reduce reactive oxygen species (ROS) | [8] |
|  | 3. *Danshen* can reduce the levels of cerebral malondialdehyde (MDA) and also can increase the activity of superoxide dismutase (SOD) in a 4-vessel occlusion rat model. | [48] |
|  | 4. ND-309 is a new metabolite of *Danshen* in rat brain, which can reduce brain tissue MDA, and also can increase the reduction of mitochondrial ATP, and mitochondrial SOD and glutathione peroxidase (GSH-Px) activities induced by ischemia-reperfusion injury. | [51] |
|  | 5. *Danshen* may reduce MDA concentration and also may increase the levels of catalase, SOD, GSH of cerebral cortex and hippocampus region in an ischemia-reperfusion injured rat model. | [52] |
